# Aspirin, salicylates and cancer: report of a meeting at the Royal Society of Medicine, London, 23 November 2010

**DOI:** 10.3332/ecancer.2011.213

**Published:** 2011-05-24

On 23 November 2010, the Aspirin Foundation held a conference at the Royal Society of Medicine in London to consider the latest evidence for the role of aspirin in cancer prevention. The conference heard from leading specialists in the fields of epidemiology, genetics and gastroenterology, who discussed the implications of recent studies and considered how the role of aspirin might develop in the near future.

The conference was chaired by Professor Peter Elwood, Honorary Professor of Epidemiology at the University of Cardiff, who published the first clinical trial evidence of aspirin’s preventative effects against cardiovascular disease [1]. He said that, over the years, the indications for aspirin have increased from the treatment of pain and fever to include prevention of cardiovascular disease and stroke and preeclampsia. Work is now underway to evaluate its role in the treatment of vascular dementia and cataracts.

Recent evidence linking aspirin use with a reduced risk of several cancers had brought medicine to ‘the brink of a breakthrough of enormous importance’, Professor Elwood added, and could change the balance of risk and benefit in favour of wider use of aspirin as prophylaxis. He suggested that aspirin use was now a question of personal responsibility for health. Evidence of the risks and benefits of taking aspirin should be presented to the public in a package of measures to preserve health, so that individuals could make an informed choice about managing their health for themselves.

## Figures and Tables

**Figure 1:**
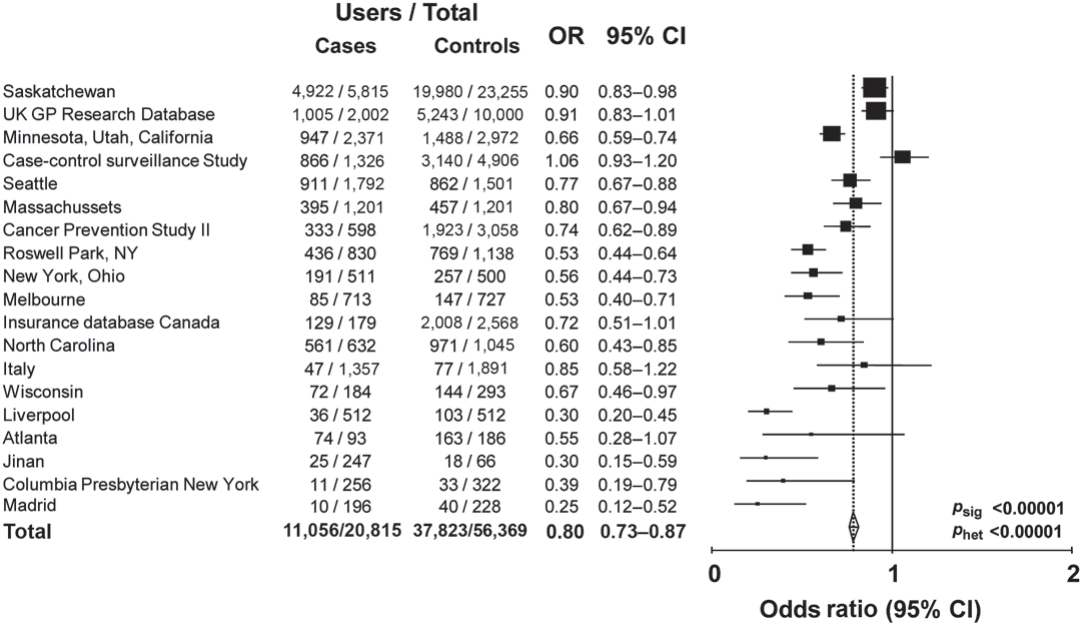
Any use of aspirin or NSAID in cases of colorectal cancer versus age and sex matched controls: 19 case–control studies.

**Figure 2:**
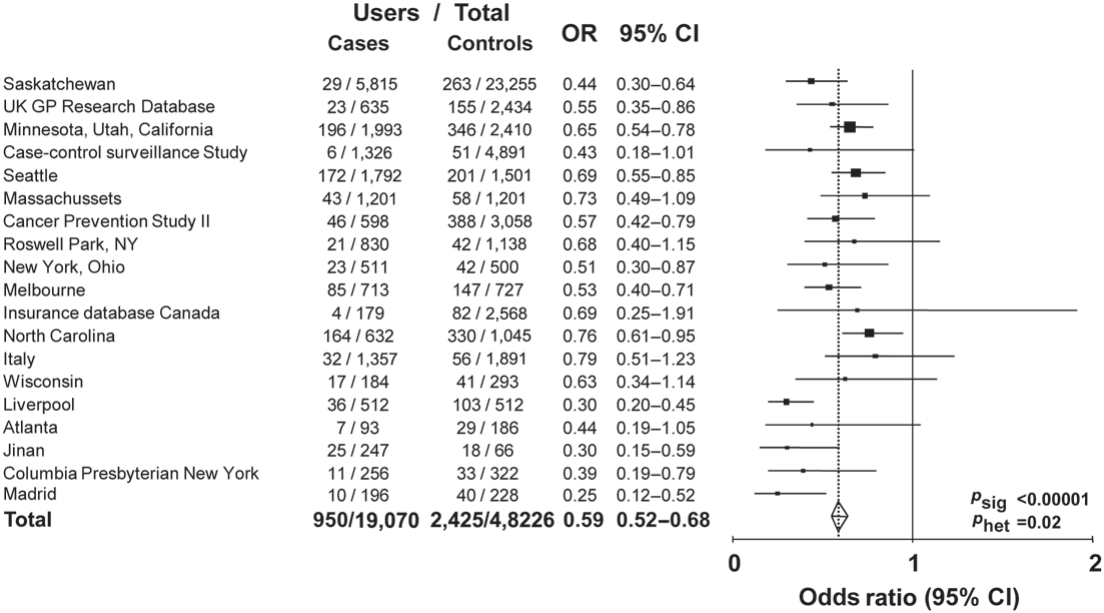
Maximum use of aspirin or NSAID in cases of colorectal cancer versus age- and sex-matched controls: 19 case–control studies.

**Figure 3:**
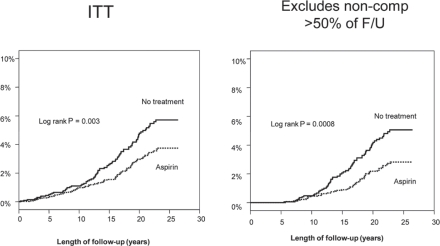
Colorectal cancer in the pooled analysis. UK-TIA cases with at least 5 years scheduled trial treatment.

**Figure 4:**
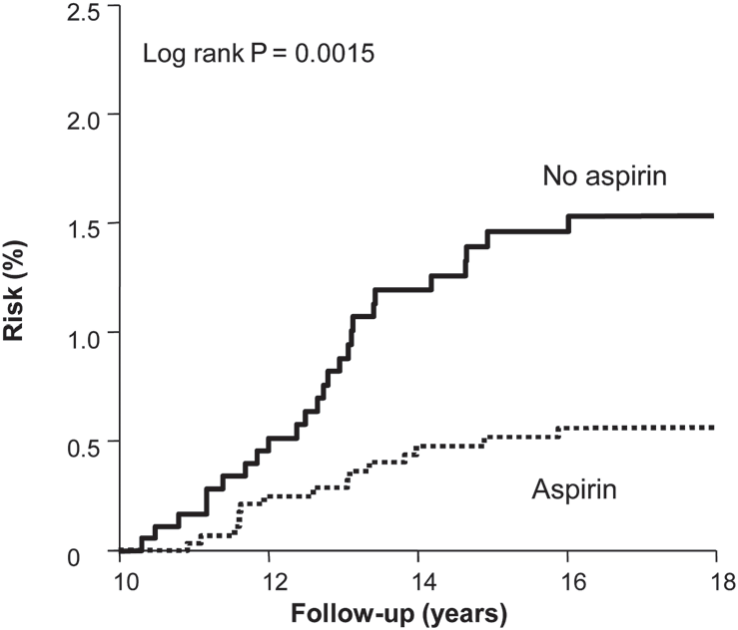
Delayed effect of aspirin on risk of colorectal cancer in the UK-TIA Trial and British Doctors Aspirin Trial in compliant patients with scheduled treatment ≥5 years.

**Figure 5:**
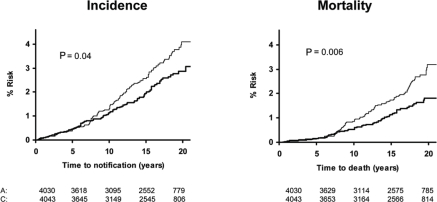
Pooled analysis of the effect of low-dose (75–300 mg) aspirin (thick line) versus control (thin line) on subsequent incidence and mortality due to colorectal cancer in TPT, SALT and UK-TIA.

**Figure 6:**
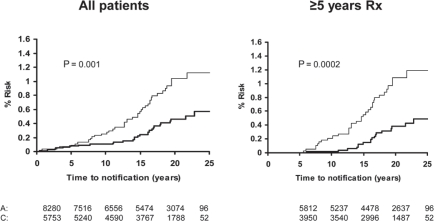
Pooled analysis of the effect of low-dose (75–300 mg) aspirin (thick line) versus control (thin line) on subsequent incidence of proximal colorectal cancer in BDAT, TPT, SALT and UK-TIA.

**Figure 7:**
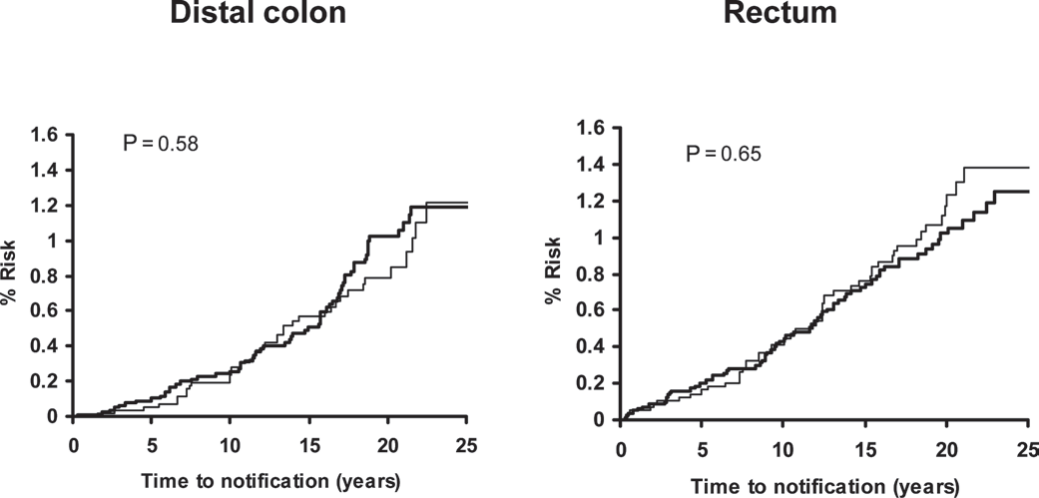
Pooled analysis of the effect of low-dose (75–300 mg) aspirin (thick line) versus control (thin line) on subsequent incidence of distal colon and rectal cancer in BDAT, TPT, SALT and UK-TIA.
