# Supplementary material for: Aspirin, salicylates and cancer: report of a meeting at the Royal Society of Medicine, London, 23 November 2010
Source: Ecancermedicalscience. 2011 May 24;5:213. doi: 10.3332/ecancer.2011.213 (PMC3223940; doi:10.3332/ecancer.2011.213)
Supplement: Related texts

# Sub article: Current evidence on aspirin and cancer prevention

**Published:** 2011

**Authors:** G Morgan

**Affiliations:** Project Manager for Older People’s Services, NHS Wales, UK

Dr Morgan’s review of the evidence of aspirin and the risk of cancers other than colorectal cancer predated the recent analysis of cancer mortality in randomised trials of aspirin [1].

The first hint that aspirin use may reduce the risk of cancer was published in 1988: an Australian observational study found a significantly lower risk of colorectal cancer among men and women who reported aspirin use [2]. However, reviews by the International Agency for Research on Cancer (IARC) in 1997 and 2001 concluded there was promising evidence of a beneficial effect from observational data but this was limited by a lack of randomised prospective trials [3,4].

More recently, studies involving a wider range of cancers have been summarised in two reviews [5,6]. These reviews identified two case–control (*n* = 2,000) and six cohort studies (*n* = 1,000) of aspirin and lung cancer risk; the case–control studies suggested that aspirin was associated with a 10–40% reduction in risk but the cohort studies were inconclusive. Randomised controlled trials have shown that aspirin reduces the risk of death from lung cancer by 20–30%. In oesophageal cancer, two case–control studies and four cohort studies (total ∼2,000 patients) found a reduced risk but wide variation in estimates of the effect size. The ASPirin Esomeprazole Chemoprevention Trial (AspECT) is currently evaluating the effect of low-dose aspirin plus esomeprazole on overall mortality and cancer conversion in patients with Barrett’s oesophagus.

Twelve case–control and six cohort studies involving ∼30,000 cases suggest that aspirin is associated with a 25% lower risk of breast cancer. The Women’s Health Study, with a total of almost 40,000 participants, failed to show that aspirin 100 mg on alternate days reduced the risk of developing breast cancer [7] but it is possible that this was too low a dose.

In stomach cancer, three case–control studies (1,600 cases) suggest a possible risk reduction of 20–40% with aspirin; four cohort studies (1,400 cases) suggest a smaller benefit but with less certainty. For pancreatic cancer, one case–control study (200 cases) found no benefit whereas five cohort studies involving ∼5,000 cases reported a small risk reduction. In ovarian cancer, six case–control studies (3,000 cases) suggest that aspirin may reduce risk but the size of this effect is unclear; one cohort study (500 cases) found no evidence of a benefit.

Approximately 30 case–control studies including 25,000 cases provide no clear evidence that aspirin is associated with a lower risk of cancers of the prostate, bladder or kidney. By contrast, 10 cohort studies involving about 15,000 men with prostate cancer suggest a small reduction in the risk of prostate cancer. Five cohort studies (4,000 cases) have identified a small increased risk of kidney cancer associated with aspirin use. Six observational studies involving 2,000 cases of lymphoma found no change in risk associated with aspirin use.

These findings raise the question of how to interpret observational evidence of aspirin and the risk of various cancers in light of strong evidence that it reduces the risk of colorectal and lung cancer. Does aspirin block pathways involved in cancer development? If it does, what level of evidence is necessary to establish its role? In 2006, a citizen’s jury considered the available evidence and called for a health education campaign about the benefits and risks of aspirin in preventing cancer and cardiovascular disease [8]. Although health policy has not changed since, a recent survey found that about a quarter of older people are taking prophylactic aspirin [9]. Health professionals must now consider how to meet the information needs of the public.

Aspirin might also be helpful in other ways in the adjuvant treatment of cancer. In cancer patients with acute coronary syndrome and thrombocytopenia, aspirin users had a 90% survival rate after one week compared with 6% in nonaspirin users [10]. In this study, no significant undesirable effects of aspirin, notably increased bleeding risk, were observed.

The aspirin metabolite salicylate induces programmed cell death in cancer cells. Aspirin may therefore be useful in combination with chemotherapy [11]. Furthermore, aspirin may reduce the adverse effects of chemotherapy, which may be partly mediated by prostaglandins [8].

Finally, as the population ages and the effectiveness of cancer treatments improves, it is likely that more people in the future will be living with cancer. Prophylaxis with aspirin may be useful in this group, given that the risk of vascular events also increases with age.

## References

[1] Rothwell PM, Fowkes FG, Belch JF (2011). Effect of daily aspirin on long-term risk of death due to cancer: analysis of individual patient data from randomised trials. Lancet.

[2] Kune GA, Kune S, Watson LF (1988). Colorectal cancer risk, chronic illnesses, operations, and medications: case control results from the Melbourne Colorectal Cancer Study. Cancer Res.

[3] Vainio H, Morgan G, Kleihues P (1997). An international evaluation of the cancer-preventive potential of nonsteroidal anti-inflammatory drugs. Cancer Epidemiol Biomarkers Prev.

[4] Morgan G, Vainio H (2002). NSAIDs and cancer prevention: a review of the recent evidence. Curr Topics Pharmacol.

[5] Bosetti C, Gallus S, La Vecchia C (2009). Aspirin and cancer risk: a summary review to 2007. Recent Results Cancer Res.

[6] Cuzick J, Otto F, Baron JA (2009). Aspirin and non-steroidal anti-inflammatory drugs for cancer prevention: an international consensus statement. Lancet Oncol.

[7] Cook NR, Lee IM, Gaziano JM (2005). Low-dose aspirin in the primary prevention of cancer: the Women’s Health Study: a randomized controlled trial. JAMA.

[8] Elwood P, Longley M (2010). My health: whose responsibility? A jury decides. J Epidemiol Community Health.

[9] Elwood P, Morgan G, Fone D, Dunstan F A survey of aspirin taking in a South Wales county.. Br J Cardiol.

[10] Sarkiss MG, Yusuf SW, Warneke CL (2007). Impact of aspirin therapy in cancer patients with thrombocytopenia and acute coronary syndromes. Cancer.

[11] Voutsadakis IA, Patrikidou A, Tsapakidis K (2010). Additive inhibition of colorectal cancer cell lines by aspirin and bortezomib. Int J Colorectal Dis.

# Sub article: Effects on colon cancer risk in randomised trials of aspirin

**Published:** 2011

**Authors:** P Rothwell

**Affiliations:** Department of Neurology, University of Oxford, Oxford, UK

Observational data show that aspirin or non-steroidal anti-inflammatory drug use is associated with a reduced risk of colorectal cancer. Analysis of 19 case–control studies involving a total of 20,815 cases and 56,369 age- and sex-matched controls shows that the risk is 20% lower for any use of these medicines [odds ratio (OR) 0.80, 95% CI 0.73–0.87) (Figure 1) and 40% lower in the subgroups with highest reported use (OR 0.59, 95% CI 0.52–0.68) (Figure 2). However, observational studies do not prove a causal relationship and are potentially misleading as a means of identifying the effects of medicines.

Several randomised trials have shown that aspirin [1–4] and selective COX-2 inhibitors [5–7] reduce the recurrence of colorectal polyps but, with a follow-up of 2–3 years and because only 10% of adenomas progress to cancer, they could not provide evidence about colorectal cancer risk. Two large randomised US trials found no evidence that aspirin reduces the risk of colorectal cancer. The Physicians’ Health Study compared aspirin 325 mg on alternate days with placebo in over 22,000 male doctors and found no risk reduction over 12 years’ follow-up [8]. In the Women’s Health Study, nearly 40,000 women were randomised to treatment with aspirin 100 mg on alternate days or placebo; after 10 years, there was no reduction in either the risk of adenomas or colorectal cancer [9].

In the UK, two large randomised trials have evaluated the effects of aspirin in preventing vascular events of aspirin have been carried out in the UK. The UK-TIA trial compared aspirin 1,200 or 300 mg/day with placebo in 2,449 men [10]; 12% of men assigned to aspirin had stopped treatment by 4 months and 12% of those assigned to placebo started aspirin during the trial. The British Doctors Aspirin Trial compared aspirin 300 or 500 mg/day with a strategy of avoiding aspirin (but not assigning a placebo) in 5,139 men [11]; 19% stopped aspirin within the first year, 5% did so in each subsequent year and 2% of controls began aspirin use each year.

A median of 23 years’ follow-up is available for both studies. Using UK cancer registration and death certificates for case ascertainment, analysis of patient data from these studies showed a significant reduction in the risk of colorectal cancer among aspirin users (hazard ratio 0.65, 95% CI 0.49–0.87) but no significant risk reduction of other cancers. The analyses by intention to treat and excluding non-compliant patients are illustrated in Figure 3. Bearing in mind the low rates of compliance in these trials, this represents a conservative estimate of the effects of aspirin. Stratifying colorectal cancer risk by 5-year periods from randomisation reveals that the onset of reduced risk is delayed by 10 years, with a duration corresponding to the length of time of aspirin use. The largest potential effect of aspirin is illustrated in Figure 4, which includes participants who were compliant at first follow-up and took aspirin for at least 5 years.

Long-term follow-up data (>20 years) are now available from three other large randomised trials of aspirin: the Thrombosis Prevention Trial (TPT; *n* = 5,085) [12], the Swedish Aspirin Low Dose Trial (SALT) (*n* = 1,360) [13] and the Dutch TIA Aspirin Trial (*n* = 2,455) [14]. The dose of aspirin in these trials was 75–300 mg/day and the duration of treatment was approximately 7 years in TPT and 2.6 and 2.7 years in the other trials.

Analysis of patient-level data from these trials and the two UK trials shows that aspirin reduced the risk of death from colorectal cancer by approximately one-third overall (OR 0.66, 95% CI 0.51–0.84) and by over 40% among participants who took low-dose aspirin for at least 2.5 years (OR 0.57, 95% CI 0.40–0.81). The effect of higher dose aspirin (500–1,200 mg/day) was of borderline statistical significance (OR 0.72, 95% CI 0.50–1.03).

Pooled analysis of the SALT, UK-TIA and TPT trials showed that low-dose aspirin reduced the incidence and mortality of colorectal cancer, with a more marked effect seen in participants treated for at least 5 years (Figure 5). In all five trials, the benefit was confined to tumours of the proximal colon (Figure 6); there was no significant reduction in the risk of distal or rectal tumours (Figure 7) though the confidence intervals were wide and an effect cannot be excluded (particularly for rectal tumours). This distinction is biologically plausible because of differences between proximal and distal colons in embryological origins, risk factors and epidemiology of tumours, and different responsiveness to treatment [15–19]. It is possible that aspirin may be more effective against non-polypoid cancers that grow more aggressively in the proximal colon.

These data suggest that taking aspirin at a dose of 75 mg/day reduces the incidence and mortality due to sporadic colorectal cancer by up to 50% after a latent period of about 7–10 years. This is followed by a gradual catch-up of rates as the effects of aspirin diminish. Confining the analysis to patients who could have taken aspirin for at least 5 years suggests a larger effect. Latency will depend on the point of action of aspirin, which might differ between clinical situations, and the effect size will depend on the accuracy of screening procedures. Follow-up of other randomised trials is required to determine the effects of lower and less frequent doses.

## References

[1] Baron JA, Cole BF, Sandler RS (2003). A randomized trial of aspirin to prevent colorectal adenomas. N Engl J Med.

[2] Sandler RS, Halabi S, Baron JA (2003). A randomized trial of aspirin to prevent colorectal adenomas in patients with previous colorectal cancer. N Engl J Med.

[3] Benamouzig R, Deyra J, Martin A (2003). Daily soluble aspirin and prevention of colorectal adenoma recurrence: one-year results of the APACC trial. Gastroenterology.

[4] Logan RF, Grainge MJ, Shepherd VC (2008). Aspirin and folic acid for the prevention of recurrent colorectal adenomas. Gastroenterology.

[5] Bertagnolli MM, Eagle CJ, Zauber AG (2006). Celecoxib for the prevention of sporadic colorectal adenomas. N Engl J Med.

[6] Arber N, Eagle CJ, Spicak J (2006). Celecoxib for the prevention of colorectal adenomatous polyps. N Engl J Med.

[7] Baron JA, Sandler RS, Bresalier RS (2006). A randomized trial of rofecoxib for the chemoprevention of colorectal adenomas. Gastroenterology.

[8] Stürmer T, Glynn RJ, Lee IM (1998). Aspirin use and colorectal cancer: post-trial follow-up data from the Physicians’ Health Study. Ann Intern Med.

[9] Cook NR, Lee IM, Gaziano JM (2005). Low-dose aspirin in the primary prevention of cancer: the Women’s Health Study: a randomized controlled trial. JAMA.

[10] Farrell B, Godwin J, Richards S (1991). The United Kingdom transient ischaemic attack (UK-TIA) aspirin trial: final results. J Neurol Neurosurg Psychiatr.

[11] Peto R, Gray R, Collins R (1988). Randomised trial of prophylactic daily aspirin in British male doctors. Br Med J (Clin Res Ed).

[12] Medical Research Council’s General Practice Research Framework (1998). Thrombosis prevention trial: randomised trial of low-intensity oral anticoagulation with warfarin and low-dose aspirin in the primary prevention of ischaemic heart disease in men at increased risk. Lancet.

[13] The SALT Collaborative Group (1991). Swedish Aspirin Low-Dose Trial (SALT) of 75 mg aspirin as secondary prophylaxis after cerebrovascular ischaemic events. Lancet.

[14] The Dutch TIA Trial Study Group (1991). A comparison of two doses of aspirin (30 mg vs 283 mg a day) in patients after a transient ischemic attack or minor ischemic stroke. N Engl J Med.

[15] Elsaleh H, Joseph D, Grieu F (2000). Association of tumour site and sex with survival benefit from adjuvant chemotherapy in colorectal cancer. Lancet.

[16] Bufill JA (1990). Colorectal cancer: evidence for distinct genetic categories based on proximal or distal tumor location. Ann Intern Med.

[17] Iacopetta B (2002). Are there two sides to colorectal cancer?. Int J Cancer.

[18] Birkenkamp-Demtroder K, Olesen SH, Sørensen FB (2005). Differential gene expression in colon cancer of the caecum versus the sigmoid and rectosigmoid. Gut.

[19] Leopoldo S, Lorena B, Cinzia A (2008). Two subtypes of mucinous adenocarcinoma of the colorectum: clinicopathological and genetic features. Ann Surg Oncol.

# Sub article: Aspirin in the prevention and treatment of hereditary colorectal cancer

**Published:** 2011

**Authors:** J Burn

**Affiliations:** Institute of Human Genetics, University of Newcastle, Newcastle upon Tyne, UK

People with familial adenomatous polyposis have APC mutations, the precursor of sporadic colon cancer, and are at high risk of developing colon cancer. They are a valuable group of patients for chemoprevention trials because they are a homogeneous population with a high incidence of disease, and they are highly motivated to participate and are already under surveillance due to their high incidence of disease. As most colorectal cancers are initiated by loss of APC function, these patients with an inherited defect are an ideal model system for sporadic cancers.

The first trial carried out by the Colorectal Adenoma/carcinoma Prevention Programme (CAPP1) evaluated the effect of aspirin and/or resistant starch to prevent polyp formation in several European centres [1]. Resistant starch is metabolised by colonic bacteria to butyrate, which has anticancer activity in vitro and in vivo [2]. Patients (mean age 18) were randomised in a factorial design to receive Hylon VII/potato starch or aspirin 600 mg/day, alone and in combination, or placebo. Endpoints were the number of adenomas (estimated from the endoscopy report and a video recording of the distal colon), changes in the size of the largest polyp on endoscopy, and markers of cell growth and proliferation in mucosal biopsy.

133 patients completed at least one year’s treatment. There was no apparent effect on adenoma numbers but this endpoint was difficult to measure in a multi-centre trial and due to the large numbers of polyps that develop in these patients [3]. By contrast, they developed too few rectal polyps to make this endpoint useful. There was a trend for patients randomised to aspirin to have smaller “biggest polyps” than in placebo cases, with a trend to significance in those treated for at least 1 year (Figure 1). Follow-up over 6 years revealed that resistant starch was associated with progressively and significantly shorter mean length of colonic crypt cells. The biological implications of this are uncertain.

Patients with Lynch syndrome, caused by an hMSH2 mutation that reduces DNA repair, are also prone to develop colon cancer (and endometrial cancer) but who tend to be older and have a lower incidence of polyps than is the case with familial adenomatous polyposis. Patients undergo colonoscopy every 1–2 years and therefore form a valuable group in which to study the effects of chemoprevention. Lynch syndrome accounts for approximately 3% of all cases of colorectal cancer and is also a good model people with microsatellite unstable cancers with chromosome stability, who represent 12% of all colorectal cancers.

The CAPP2 trial repeated the CAPP1 design in 1,071 people with Lynch syndrome recruited from 41 centres worldwide. Treatment was administered as 600 mg enteric-coated aspirin and/or 30g of a proprietary resistant starch (Novelose) or placebo cornstarch. After follow-up of up to 4 years and a mean treatment duration of 29 months, there was no difference in the incidence of adenoma or cancer of the colon, irrespective of treatment arm [4]. The study was designed to continue double blind follow-up for up to 10 years post randomisation. This revealed that aspirin was associated with a lower incidence of colorectal cancers compared with placebo, with a divergence beginning after ∼4 years (coinciding with the end of the intervention phase) (Figure 2). This association is also evident when all cancer outcomes are included in the analysis (Figure 3). Per protocol analysis confined to participants who completed 2 years of treatment demonstrated that aspirin use approximately halved the risk of developing any cancer associated with Lynch syndrome after up to 11 years’ follow-up (publication pending). There were 11 episodes of bleeding among aspirin recipients in the intervention phase and 9 with placebo; the number of strokes or myocardial infarctions was one with aspirin and 5 with placebo.

Colorectal tumours in patients with Lynch syndrome are less likely to have increased expression of COX-2, raising the question of how aspirin is reducing the risk of cancer. Aspirin appears to have an indirect effect on cancer cells with a latency of several years before the effect becomes apparent. One possibility is that aspirin promotes apoptosis in aberrant stem cells in colon crypts, which is the mechanism by which salicylates protect plants in the presence of infection. Another hypothesis is that aspirin renders crypt stem cells more susceptible to the immune system via inhibition of interleukin 4. The latent period for most people appears to be about 10 years but for individuals with Lynch syndrome, where disease progression is much faster, the latent period may be as short as 4 years.

A third CAPP study is now being planned. This will be a dose-finding study of enteric-coated aspirin 100 versus 600 mg/day for 5 years in 2,500 MMR gene carriers, using web-based recruitment. Follow-up will be at least 10 years. DNA will banked to allow identification of modifiers of response.

## Figures and Tables

**Figure 1:** CAPP1 trial: patients randomised to aspirin tended to have smaller ‘biggest polyps’ versus placebo, with a trend to significance in those treated for at least one year.

**Figure 2:** Post trial divergence of colorectal cancer incidence in the CAPP2 trial, aspirin versus placebo (*n* = 46).

**Figure 3:** Lifetable analysis, time to first ‘Lynch Syndrome cancers’ (colorectal and endometrial) in the CAPP2 trial. Hazard ratio 0.62 (95% CI 0.41–0.96), *P* = 0.03.

## References

[1] Mathers JC, Mickleburgh I, Chapman PC (2003). Can resistant starch and/or aspirin prevent the development of colonic neoplasia? The Concerted Action Polyp Prevention (CAPP) 1 Study. Proc Nutr Soc.

[2] Williams EA, Coxhead JM, Mathers JC (2003). Anti-cancer effects of butyrate: use of micro-array technology to investigate mechanisms. Proc Nutr Soc.

[3] Burn J, Bishop DT, Chapman PD (2011). A Randomized Placebo-Controlled Prevention Trial of Aspirin and/or Resistant Starch in Young People with Familial Adenomatous Polyposis. Cancer Prev Res (Phila).

[4] Burn J, Bishop DT, Mecklin JP (2008). Effect of aspirin or resistant starch on colorectal neoplasia in the Lynch syndrome. N Engl J Med.

# Sub article: Mode of action and dosage of aspirin

**Published:** 2011

**Authors:** A Chan

**Affiliations:** Gastrointestinal Unit, Massachusetts General Hospital and Harvard Medical School, Boston, Massachusetts, USA

Understanding of how aspirin reduces the risk of cancer is incomplete. Possible mechanisms include inhibition of nuclear factor-κB, induction of p38 kinase and catabolism of polyamines but most research has focused on the inhibition of COX-2 and this has the strongest evidence base. COX-2 is over-expressed by some cancers, including colorectal cancer (Figure 1) [1]. These mechanisms are not exclusive and may be synergistic.

This hypothesis was tested by analysis of molecular markers in two large cohort studies providing long-term data on aspirin use: the Nurses’ Health Study (*n* = 121,700), which began in 1976, and the Health Professionals Follow-up Study (*n* = 51,539), which began in 1986. Both studies found that aspirin was associated with an ∼30% reduced risk of colon cancer, which is consistent with the evidence of randomised trials.

Tumour specimens were collected from both cohorts from the late 1990s, representing 662 cases (58%) in the Nurses’ Health Study and 648 (76%) in the Health Professionals Follow-Up Study. Analysis was limited to 368 and 268 cases respectively for which samples of sufficient tumour tissue and adjacent normal tissue allowed comparison using positive and negative controls. Figure 2 illustrates how immunostaining identifies tumours that are negative (A and B) and positive (C and D) for COX-2.

Overall, these two studies showed that regular aspirin use was associated with a lower risk of colorectal cancer (relative risk, RR, 0.73; 95% CI 0.62–0.86). However, this benefit was largely confined to individuals whose tumour stained positive for COX-2 (RR 0.64, 95% CI 0.52–0.78) and not those with COX-2 negative tumours (RR 0.96, 95% CI 0.73–1.26) [2]. This finding suggests that COX-2 positive status may have potential as a marker of response to aspirin.

Patients diagnosed with colorectal cancer are a high-risk group for whom aspirin therapy may be useful. In these studies, 1,279 participants (mean age 65) were diagnosed with stage I, II or III colorectal cancer and underwent curative resection. After a median follow-up of 11.8 years there were 480 total deaths and 222 deaths due to colorectal cancer. Aspirin use before diagnosis was not associated with colorectal cancer-specific survival (HR 1.05, 95% CI 0.80–1.37) or overall survival (HR 0.93, 95% CI 0.77–1.11) [3]. This is not surprising because colon cancer was developing in these individuals despite their use of aspirin. By contrast, aspirin use after diagnosis was associated with a significant reduction in colorectal cancer-related death and overall mortality (Figure 3). This effect was more marked among participants who had not used aspirin before diagnosis (multivariate relative risk 0.53, 95% CI 0.33–0.86) compared with those who had (multivariate relative risk 0.89, 95% CI 0.59–1.35); and in those with COX-2 positive tumours (multivariate relative risk 0.39, 95% CI 0.20–0.76) compared with COX-2 negative tumours (multivariate relative risk 1.22, 95% CI 0.36–4.18). There was a strong dose-response relationship between aspirin (represented by number of 325 mg tablets taken per week) and the relative risk of adenoma (Figure 4) [4] and colorectal cancer in men and women (Figures 5 and 6) [5,6]. Secondary surveys demonstrated that people reporting consumption of 7–14 tablets per week were actually taking aspirin daily. These figures challenge the belief that low-dose aspirin provides sufficient protection against colorectal cancer and further studies are needed to clarify the minimum effective dose. This is supported by the Cancer Prevention Study II, which found greater risk reductions for colorectal, prostate and breast cancers with high versus low doses and longer use (<5 versus >5 years) of aspirin [7].

In these cohorts with ∼20 years of follow-up, 11 deaths occurred due to gastrointestinal bleeding. The relative risk of major gastrointestinal bleeding (requiring a blood transfusion or hospitalisation for bleeding) was lower than has been reported in randomised trials of aspirin for secondary prevention of cardiovascular events (Figure 7). This may have been because these cohorts had lower morbidity than trial participants but the risk of major bleeding is attenuated with increasing duration of use and aspirin consumption by these cohorts was longer than in clinical trials, due either to adaptation of the gastrointestinal mucosa or treatment discontinuation when adverse events occur. These data also suggest that there is little difference in bleeding risk between low and higher doses of aspirin.

## Figures and Tables

**Figure 1:** Expression of COX-2 by normal gastrointestinal cells, adenoma and colon cancer (adapted from Eberhart et al [1].).

**Figure 2:** Immunostaining for COX-2 (dark brown areas) in colorectal tumours. Samples A and B are negative for COX-2; samples C and D are positive for COX-2.

**Figure 3:** Aspirin use after diagnosis and colorectal cancer-related and overall survival [3] (A) Colorectal cancer-related mortality (log rank *P* = 0.02). (B) Overall mortality (log rank *P* = 0.03).

**Figure 4:** Dose of aspirin and risk of adenoma [4].

**Figure 5:** Dose of aspirin and risk of colorectal cancer in women [6].

**Figure 6:** Dose of aspirin and risk of colorectal cancer in men.

**Figure 7:** Relative risk of major gastrointestinal bleeding and dose of aspirin in the Nurses’ Health Study and Health Professionals Follow-up Study cohorts.

## References

[1] Eberhart CE, Coffey RJ, Radhika A (1994). Up-regulation of cyclooxygenase 2 gene expression in human colorectal adenomas and adenocarcinomas. Gastroenterology.

[2] Chan AT, Ogino S, Fuchs CS (2007). Aspirin and the risk of colorectal cancer in relation to the expression of COX-2. N Engl J Med.

[3] Chan AT, Ogino S, Fuchs CS (2009). Aspirin use and survival after diagnosis of colorectal cancer. JAMA.

[4] Chan AT, Giovannucci EL, Schernhammer ES (2004). A prospective study of aspirin use and the risk for colorectal adenoma. Ann Intern Med.

[5] Chan AT, Giovannucci EL, Meyerhardt JA (2005). Long-term use of aspirin and nonsteroidal anti-inflammatory drugs and risk of colorectal cancer. JAMA.

[6] Chan AT, Giovannucci EL, Meyerhardt JA (2008). Aspirin dose and duration of use and risk of colorectal cancer in men. Gastroenterology.

[7] Jacobs EJ, Thun MJ, Bain EB (2007). A large cohort study of long-term daily use of adult-strength aspirin and cancer incidence. J Natl Cancer Inst.

# Sub article: Aspirin, salicylates and cancer: Salicylate salts in plants

**Published:** 2011

**Authors:** L Mur

**Affiliations:** Deputy Leader of Bio-renewables and Environmental Change Research Division, Aberystwyth University, Aberystwyth, UK

Plant sources of salicylates have been used for medicinal applications for thousands of years. The Egyptians used extracts of myrtle or willow leaves for joint pain and Hippocrates recommended chewing willow leaves to provide analgesia during childbirth. Edward Stone is credited with identifying salicylin as the active component of willow bark in the 18th century; willow bark is still used as a herbal medicine today. The concentration of salicylin is very high in willow, where it is presumed to act as an antifeedant [1]. It is present at lesser levels in every plant species and is readily metabolised to the more active forms salicylic acid and methyl salicylate; acetylated salicylate (aspirin) is not present in plants. Although plants do not have cyclo-oxygenases it is likely that the multiple roles played by salicylates may shed light on possible mechanisms of action that could be relevant to man.

Classically, salicylates have been associated with regulating defence against pathogens but they are now emerging as multifunctional hormones protecting plants against stress. They are involved in signalling and the plant’s response to stressors such as disease, cold and warming and in senescence. In disease, aalicylic acid plays a dual role: it facilitates the lethal effects of pathogens that kill the plant host in order to obtain nutrients (necrotrophs) but inhibits and confines the effects of pathogens that rely on plant survival (biotrophs) by promoting programmed cell death (PCD) as a means of confining the spread of disease. In plants PCD, is triggered by recognition of the pathogen which initiates a kinase-dependent signalling cascade leading to the generation of oxygen free radicals and nitric oxide. The synthesis of salicylic acid is increased within 4–5 hours of infection which promoting defence gene expression and PCD [2]. Salicylic acid is also distributed throughout the plant to promote systemic acquired resistance genes, conferring protection against a wide range of pathogens [3].

Additionally, a plant can evolve methyl salicylate into its environment and confer inter-plant resistance. Methyl salicylate is produced in response to an attack by predators such as greenfly; this attracts insects such as hover flies and ladybirds which feed on greenfly.

The generation of transgenic plants encoding the bacterial gene – salicylate hydroxylase (NahG) – which reduces salicylate levels has provided great insights have shown the importance of salicylate to other forms stress, for example, tolerance to heat and chilling is compromised in these plants. Salicylic acid increases production of heat shock proteins, which act as chaperone proteins to protect unfolded proteins from heat [4]. When a plant is chilled, salicylic acid suppresses growth by increasing cross-linkage in the cell wall, limiting expansion [5].

At a molecular level, NahG plants have indicated the salicylate regulates a plethora of plant genes but other features have direct relevance for salicylate action in humans. Specifically, salicylic acid (at microgram levels) acts to potentiate the activity of NADPH oxidase, which increase oxygen free radical generation and perturbs mitochondrial function [2]. This causes an oxidative burst that kills the pathogen There is some limited evidence that a similar potentiation of NADPH oxidase may occur in humans [6]. Unusually in plant biochemistry, salicylate promotes the release of cytochrome C from the mitochondrion which in man, C triggers the formation of apoptosomes, though there is no equivalent in plants and its role is uncertain [2]. This raises the possibility that salicylate could play an important role in triggering apoptosis in humans [6].

Salicylate levels are higher in plants that are exposed to stresses such as infection and temperature change than those cultivated in protected environments like greenhouses. This suggests that much of the food we grow may have low levels of salicylates. It is conceivable that humans benefitted from the protective effects of plant-derived salicylates when their diet comprised wild plants. Conversely, it is likely that a modern diet relying mass-produced plants lacks salicylate and, possibly, is less beneficial. However, salicylates also have industrial applications: they may be added artificially to improve a plant’s resistance to the effects of chilling and some fungicides act partly by increasing plant salicylate levels.

## References

[1] Wagner S, Ureña A, Reich E (2008). Validated HPTLC methods for the determination of salicin in Salix sp and of harpagoside in Harpagophytum [corrected] procumbens. J Pharm Biomed Anal.

[2] Mur LA, Kenton P, Lloyd AJ (2008). The hypersensitive response; the centenary is upon us but how much do we know?. J Exp Bot.

[3] Ward ER, Uknes SJ, Williams SC (1991). Coordinate Gene Activity in Response to Agents That Induce Systemic Acquired Resistance. Plant Cell.

[4] Clarke SM, Mur LA, Wood JE (2004). Salicylic acid dependent signaling promotes basal thermotolerance but is not essential for acquired thermotolerance in Arabidopsis thaliana. Plant J.

[5] Scott IM, Clarke SM, Wood JE (2004). Salicylate accumulation inhibits growth at chilling temperature in Arabidopsis. Plant Physiol.

[6] Chung YM, Bae YS, Lee SY (2003). Molecular ordering of ROS production, mitochondrial changes, and caspase activation during sodium salicylate-induced apoptosis. Free Radic Biol Med.

# Sub article: Colon cancer screening programmes and the place for aspirin

**Published:** 2011

**Authors:** D Morton

**Affiliations:** Professor of Colorectal Surgery, University of Birmingham, Birmingham, UK

Survival in patients with colorectal cancer has been increasing over the last 50 years (Figure 1), largely due to improvements in diagnosis and management. Whereas clinicians once sought primarily to diagnose disease in symptomatic patients they are now monitoring an at-risk population, some of whom will develop colorectal cancer.

Data from a faecal occult blood screening programme demonstrates the earlier detection of colorectal cancer (Table 1). The Dukes staging system grades colorectal cancer from A (essentially curable) to D (metastatic). The distribution of stages in an unscreened population is similar to that among people who do not respond to invitation for screening (column 4): almost a third of non-responders have metastatic disease and few have Stage A disease. With screening, over half of patients are diagnosed with Stage A disease and the proportion with metastatic disease is greatly reduced.

Increased screening has resulted in a huge shift in the stage of disease at diagnosis and in outcome: 2-yearly faecal occult blood screening reduces colorectal cancer mortality by 15% and increases the proportion of cancers diagnosed at an early stage [1,2].

We now have the opportunity to prevent colorectal cancer. The recent UK Flexible Sigmoidoscopy trial demonstrated the benefits of detection and removal of adenomas [3]. 170,000 people aged 55–64 were randomised to undergo once-only sigmoidoscopy or to be a silent control. Compliance was 70% and median follow-up was 11 years. Intent to treat analysis showed that the incidence of colorectal cancer was reduced by 22% and mortality was reduced by 31%. Per protocol analysis showed that, in adherent patients, incidence was reduced by 33% and mortality by 44%. The number needed to treat (i.e. screen) to prevent one cancer was 191. A pragmatic interpretation of this evidence suggests that, assuming a 50% compliance, faecal occult blood screening would identify 20,000 patients with adenomas annually, and flexible sigmoidoscopy would identify 50,000 new patients annually.

Screening can define four groups according to their risk of developing colorectal cancer. Individuals with colorectal cancer and high-risk adenomas are at greatest risk. It is important to reduce the risk of recurrent tumours in these groups, for which aspirin has a role to play. Those with low-risk adenomas or no adenomas are at least risk, though within these groups are an unknown number of individuals at high risk but who cannot be detected. It may be possible to identify these individuals by genetic screening. Genome-wide association studies have shown that the risk of colorectal cancer increases with the number of known risk alleles present [4–8], reaching a 2–4-fold increased risk with 14 or more alleles. Provided these individuals were also more likely to develop adenomas, surveillance by colonoscopy would avoid the development of cancer. An unpublished analysis of 900 patients in the APPROVE trial [9] identified 7 single nucleotide polymorphisms (SNPs) strongly associated with adenoma risk; this suggests that people carrying high-risk alleles are also at increased risk of developing adenomas. Further, these individuals are likely to benefit from the therapeutic interventions currently provided for colorectal cancer.

This evidence makes a strong case for introducing chemoprevention of adenoma and colorectal cancer in high-risk individuals. There are now four randomised trials of aspirin as chemoprevention, involving a total of almost 5,000 patients; all have demonstrated a reduction in adenoma of 20–35%. This is overwhelming evidence that aspirin will prevent the formation of adenomas. The fact that aspirin reduces the risk of colorectal cancer with a latency of 10 years [10] indicates that adenoma prevention, rather than down-staging cancer or preventing progression, is probably the mechanism involved. Based on our knowledge of its safety profile, aspirin is therefore the chemoprevention agent of choice. It may be possible to increase the effectiveness of aspirin with adjunctive agents such as calcium (for which there is evidence from a randomised controlled trial [11]), vitamin D [12], selenium [13] and difluoromethylornithine [14].

It is feasible to conduct a trial to identify genetic and environmental interactions for colorectal risk assessment and prevention. A suitable design would be a cross-sectional cohort study involving 20,000 people embedded within the screening programme. This could define the effect of known and novel genomic factors associated with increased colorectal neoplasia risk through their influence on adenoma occurrence. Within this, a nested randomised controlled trial of 6,000 people could determine the combined impact of aspirin and nutritional supplements in reducing adenoma recurrence. A biosample collection programme would facilitate genomic studies.

An algorithm for this design is illustrated in Figure 2. In such a trial, 20,000 participants would be identified within the screening programme by faecal occult blood testing or colonoscopy and then undergo risk stratification. All would be entered into the genomic study but individuals with no or low-risk adenomas would not undergo randomisation. Those with high-risk adenoma who accepted aspirin treatment and consented to a trial would be randomised to adjunctive treatment, each compared with placebo. Individuals with cancer could also undergo randomisation though this may complicate interpretation of the trial.

## Figures and Tables

**Figure 1:** United Kingdom 1950–2003: males and females colorectal cancer mortality at ages 35–69.

**Figure 2:** Study design for evaluating the efficacy of aspirin plus adjunctive therapy in adenoma prevention and targeting therapy.

**Table 1:** Faecal occult blood screening increases detection of early stage (Dukes’ A) colorectal cancer [1]

**Dukes**	**First invite (%)**	**Rescreen (%)**	**Non-responder (%)**	**Interval (%)**
A	51%	37%	11%	16%
B	20%	36%	35%	31%
C	24%	18%	22%	29%
D	5%	6%	29%	24%
Total (no.)	83	132	400	249

## References

[1] Hardcastle JD, Chamberlain JO, Robinson MH (1996). Randomised controlled trial of faecal-occult-blood screening for colorectal cancer. Lancet.

[2] Kronborg O, Fenger C, Olsen J (1996). Randomised study of screening for colorectal cancer with faecal-occult-blood test. Lancet.

[3] Atkin WS, Edwards R, Kralj-Hans I (2010). Once-only flexible sigmoidoscopy screening in prevention of colorectal cancer: a multicentre randomised controlled trial. Lancet.

[4] Broderick P, Carvajal-Carmona L, Pittman AM (2007). A genome-wide association study shows that common alleles of SMAD7 influence colorectal cancer risk. Nat Genet.

[5] Tomlinson I, Webb E, Carvajal-Carmona L (2007). A genome-wide association scan of tag SNPs identifies a susceptibility variant for colorectal cancer at 8q2421. Nat Genet.

[6] Zanke BW, Greenwood CM, Rangrej J (2007). Genome-wide association scan identifies a colorectal cancer susceptibility locus on chromosome 8q24. Nat Genet.

[7] Tomlinson IP, Webb E, Carvajal-Carmona L (2008). A genome-wide association study identifies colorectal cancer susceptibility loci on chromosomes 10p14 and 8q233. Nat Genet.

[8] Houlston RS, Webb E, Broderick P (2008). Meta-analysis of genome-wide association data identifies four new susceptibility loci for colorectal cancer. Nat Genet.

[9] Baron JA, Sandler RS, Bresalier RS (2006). A randomized trial of rofecoxib for the chemoprevention of colorectal adenomas. Gastroenterology.

[10] Flossmann E, Rothwell PM, British Doctors Aspirin Trial and the UK-TIA Aspirin Trial (2007). Effect of aspirin on long-term risk of colorectal cancer: consistent evidence from randomised and observational studies. Lancet.

[11] Shaukat A, Scouras N, Schünemann HJ (2005). Role of supplemental calcium in the recurrence of colorectal adenomas: a metaanalysis of randomized controlled trials. Am J Gastroenterol.

[12] Grau MV, Baron JA, Sandler RS (2003). Vitamin D, calcium supplementation, and colorectal adenomas: results of a randomized trial. J Natl Cancer Inst.

[13] Bjelakovic G, Nikolova D, Simonetti RG, Gluud C (2008). Antioxidant supplements for preventing gastrointestinal cancers. Cochrane Database Syst Rev.

[14] Meyskens FL, McLaren CE, Pelot D (2008). Difluoromethylornithine plus sulindac for the prevention of sporadic colorectal adenomas: a randomized placebo-controlled, double-blind trial. Cancer Prev Res (Phila).

# Sub article: Aspirin and other NSAIDs for cancer prevention

**Published:** 2011

**Authors:** J Cuzick

**Affiliations:** Cancer Research UK Centre for Epidemiology, Mathematics and Statistics, London, UK

It has been almost 30 years since a reduction in adenoma formation after administration of the non-steroidal anti-inflammatory drug (NSAID) sulindac was first reported [1] and 20 years since it was confirmed in a randomised trial [2]. There is now consistent evidence from observational studies and randomised trials that aspirin and other NSAIDs reduces the risk of developing colorectal cancer and adenomas. Evidence from clinical trials also demonstrates a striking effect of combining an NSAID (sulindac) with difluoromethylornithine, which has been shown to reduce the incidence of adenomas by up to 70% compared with placebo [3]. Combinations of NSAIDs with other agents therefore offers the best approach to minimising risk, especially for high risk individuals, where toxicity is a lesser concern.

Observational studies also show that aspirin and NSAIDs are associated with reduced mortality in patients with colorectal adenoma [4,5]. This is inconsistent with the 10-year latency in reducing colorectal cancer risk that has been identified in observational and interventional studies and that has been attributed to an effect on adenomas, and suggests the possibility of two distinct mechanisms of action.

There is increasing evidence from case–control studies, though less strongly from cohort studies, that use of aspirin or NSAIDs over about 20 years is associated with a reduced risk of oesophageal cancer (Figure 1), with less conclusive evidence of a lower risk of stomach cancer(Figure 2) [6]. NSAIDs do not appear to reduce the risk of lung cancer, with case control studies suggesting a marginal effect and no reduction detected in cohort studies (Figure 3). There is accumulating evidence of a benefit in hormone-dependent cancers such as breast (Figure 4); the effect size appears to be smaller in more recent studies, suggesting the possibility of early publication bias. A recent multivariate analysis of the Nurses’ Health Study found a 40–60% reduction in the risk of recurrence among women who, after being diagnosed with breast cancer, took aspirin for 2–7 days per week [7]. Although it is not possible to determine the possible of bias in this study, this represents a greater benefit from a less expensive drug compared with current treatments. Trials are now being planned to determine whether these effects can be replicated in prospective randomised studies. Evidence of a benefit against ovarian cancer is less clear (Figure 5).

The traditional approach to phase I, II and III trials for evaluating new treatments is expensive (trials cost $10–100 million each) and may not be appropriate for evaluating preventative therapies. Instead, phase I (discovery) could rely on cohort studies and the identification of biomarkers and precursor lesions. Case–control studies are less reliable than cohort studies due to their retrospective nature and they should be carried out in carefully selected groups of patients.

Phase II studies for early validation should be improved. It is important that these studies are randomised because of the biases inherent in cohort studies. Their cost can be minimised by using biomarkers and precursor lesions as endpoints, and recruiting patients with cancer. The value of this approach is illustrated by studies of the prevention of contralateral breast tumours in women taking tamoxifen after diagnosis of breast cancer, which demonstrated a similar effect 15–20 years before it was shown in randomised trials. In the case of aromatase inhibitors, the effects on contralateral tumours suggest a 75% reduction in recurrence risk. If this strategy is shown to provide a reliable estimate of the outcomes of randomised trials, it may become the standard in the future.

Phase III studies (late definitive validation) take 10–20 years to complete and cost hundreds of millions of dollars. They are unlikely to be funded for inexpensive drugs such as aspirin, so any that are carried out must be carefully designed and use cancer mortality or incidence as an endpoint.

## Figures and Tables

**Figure 1:** NSAIDs – oesophageal cancer [6].

**Figure 2:** NSAIDs – stomach cancer [6].

**Figure 3:** NSAIDs – lung cancer [6].

**Figure 4:** NSAIDs – breast cancer [6].

**Figure 5:** NSAIDs – ovarian cancer [6].

## References

[1] Waddell WR, Loughry RW (1983). Sulindac for polyposis of the colon. J Surg Oncol.

[2] Nugent KP, Farmer KC, Spigelman AD (1993). Randomized controlled trial of the effect of sulindac on duodenal and rectal polyposis and cell proliferation in patients with familial adenomatous polyposis. Br J Surg.

[3] Meyskens FL, McLaren CE, Pelot D (2008). Difluoromethylornithine plus sulindac for the prevention of sporadic colorectal adenomas: a randomized placebo-controlled, double-blind trial. Cancer Prev Res (Phila).

[4] Zell JA, Ziogas A, Bernstein L (2009). Nonsteroidal anti-inflammatory drugs: effects on mortality after colorectal cancer diagnosis. Cancer.

[5] Chan AT, Ogino S, Fuchs CS (2009). Aspirin use and survival after diagnosis of colorectal cancer. JAMA.

[6] Bosetti C, Gallus S, La Vecchia C (2009). Aspirin and cancer risk: a summary review to 2007. Recent Results Cancer Res.

[7] Holmes MD, Chen WY, Li L (2010). Aspirin intake and survival after breast cancer. J Clin Oncol.

# Sub article: Commentary

**Published:** 2011

**Authors:** G McVie

**Affiliations:** Senior Consultant, Insituto Europeo di Oncologia, Italy

It is important to consider aspirin’s mechanism of action in the context of what we know about carcinogenesis. The idea that aspirin has at least two mechanisms of action is not new but the hypothesis that its effects on cancer risk are due to inhibition of COX-2 is less plausible than an effect on inflammation. It is therefore surprising that longitudinal studies have not been conducted in patients with inflammatory bowel disease. Studies on breast cancer stem cells show that DNA repair is important in carcinogenesis but this has not been discussed with respect to the effects of aspirin. The study proposed by Professor Dion could include a combination of aspirin with a poly (ADP-ribose) polymerase 1 (PARP1) inhibitor (PARP1 is involved in cell proliferation and differentiation and DNA repair). While the use of such a compound is not appropriate in healthy individuals, the balance of risk and benefit may be acceptable in individuals at high risk.

Another cohort that is potentially interesting is patients who have developed autoimmune colitis after treatment with ipilimumab, a T-cell suppressor. Combinations of aspirin with other treatments are another avenue that is well worth exploring in clinical trials. Vitamin D was recently shown to reduce all-cause death [1]. This would be a suitable endpoint in randomised trials of aspirin, given its effects on cardiovascular disease and cancer, though it should be acknowledged that trials are unlikely to attract large funding. In the future, trials should be randomised, smaller in scale and carried out in better-defined populations.

We need to identify early markers of outcome and it is important that we improve the collection of samples by biobanks. At the moment we do not know what tissue will be informative but the librarianship of potentially relevant tissues is essential for future research. Nor do we know the initial COX status of people who develop cancers subsequently found to be COX-2 positive or negative. Activity should focus on people who are participating in studies, whether observational or interventional, so that early markers of risk can be identified.

Several issues remain unresolved. What is the dose–response relationship in the prevention of colorectal cancer? From what we know of plant biochemistry, the effects of salicylate depend on its co ncentration. Little has been said about the effect of enteric-coating: bearing in mind its different effects on the left and right colon cancer, though there is evidence from the literature that COX2 levels are higher in tumours of the distal colon and rectum than proximal colon. It would be interesting to measure how much aspirin from an enteric-coated preparation reaches the sigmoid junction.

The aetiology of adenomas and colorectal cancer is uncertain and new therapeutic targets should be considered. The effects of aspirin on mitochondrial crypts are interesting: many treatments for parasitic diseases target parasite mitochondria but this mechanism has not been explored on clinical oncology. Molecular targets are also being identified: trastuzumab, a monoclonal antibody against human epidermal growth factor receptor 2, has recently been shown to be effective in the treatment of stomach and oesophageal cancers.

## References

[1] Autier P, Gandini S (2007). Vitamin D supplementation and total mortality: a meta-analysis of randomized controlled trials. Arch Intern Med.
